# Data on PKO biodiesel production using CaO catalyst from Turkey bones

**DOI:** 10.1016/j.dib.2018.05.103

**Published:** 2018-05-23

**Authors:** A.A. Ayoola, O.S.I. Fayomi, I.F. Usoro

**Affiliations:** a Chemical Engineering Department, Covenant University, Ota, Nigeria; b Mechanical Engineering Department, Covenant University, Ota, Nigeria; c Surface Engineering Research Centre, Tshwane University of Technology, South Africa

**Keywords:** Biodiesel, Calcined waste turkey bone, Catalyst, Palm kernel oil

## Abstract

In this research paper the production of biodiesel from palm kernel oil (PKO) using CaO obtained from waste turkey bones (WTB) and analytical grade calcium oxide was investigated. Treated WTB was reduced to fine particulate size of <150 µm and then calcinated at 800 °C for 3 h to increase its catalytic activity by its conversion from Calcium phosphate hydroxide (Ca_10_P_6_O_26_H_2_) to CaO. X-ray diffraction (XRD) and X-ray fluorescent (XRF) analysis of the analytical grade CaO, uncalcined and calcined WTB were carried out to establish their elemental chemical composition. The transesterification reaction between the triglyceride of palm kernel oil (PKO) and methanol was carried out at a constant agitation speed of 600 rpm and temperature of 65 °C, with varied methanol to oil molar ratio (8–14), catalyst concentration (1–7 wt/wt%) and the reaction time (1–3 h). Minitab 17 software (using response surface method) was employed for the design of experiment and statistical analysis required in the transesterification process of biodiesel production. The qualities of the biodiesel produced were assessed and the results obtained showed conformity of the biodiesel produced to the ASTM standard for biodiesel.

**Specifications Table**

**Table:** 

Subject area	*Materials Science Engineering*
More specific subject area	*Renewable Energy*
Type of data	*Table, image*
How data was acquired	XRF and XRD spectroscopy principles were employed in the elemental chemical analysis of CaO (analytical grade), uncalcined and calcined turkey bone used as catalyst. Biodiesel production through transesterification process (using Minitab 17, Box Benkhen design) was employed in generating data on the effects of the process parameters (main and interaction effects) on biodiesel yields. Analytical tests to determine the properties of the biodiesel obtained were carried out.
Data format	Raw, Analyzed
Experimental factors	Methanol/oil mole ratio (8–14), catalyst concentration (1–7 wt/wt%) and reaction time (1–3 hours) were the process parameters that were considered during biodiesel production (transesterification process).
Experimental features	X-ray Diffraction (XRD) analysis was carried out to determine the elemental components of the different catalyst used. The analysis involved Mac science X-ray diffraction system (MXP3A-HF) with CuKα X-ray source (λ=0.15 nm and k=1.5406 Å) operated at 30 mA and 40 kV. The diffractograms were recorded in the 2 h ranges of 5–70 with a 2 h step size of 0.03. This was done to determine the diffraction pattern of the finely grounded calcined and uncalcined waste turkey bones (WTB) catalyst. X-ray fluorescence (XRF) (Thermo Scientific ARL OP-TIM’X 166) gave the composition of both the calcined and uncalcined catalyst (WTB). That is, the elemental composition (in percent) of both the calcined and uncalcined WTB were determined.
Data source location	Department of Chemical Engineering, Covenant University, Ota, Nigeria and Metallurgical and Chemical Engineering Department, Amadu Bello University, Zaria, Kaduna State, Nigeria.
Data accessibility	Data are available within this article

**Value of the data**

• The data on biodiesel production was modelled to established the correlation and relationship between the process variables (methanol/oil mole ratio, catalyst concentration and reaction time) and the yields of biodiesel.
• The given data will show authors in the field of material science and chemical engineering that the calcination of WTB (waste turkey bone) will aid conversion of Calcium phosphate hydroxide (Ca_10_P_6_O_26_H_2_) to CaO catalyst for biodiesel production.
• The data will aid in the establishment of the optimum conditions for the production of PKO biodiesel yield.
• The models obtained from the transesterification of PKO can be used to predict the yield, operating conditions using any other vegetable oils.
• The data reveals that calcined WTB catalyst is a potential source of CaO heterogenous catalyst that can be used in the place of the conventional CaO catalyst during the transesterification of vegetable oil.

## 1 Data

**Fig. 1:**
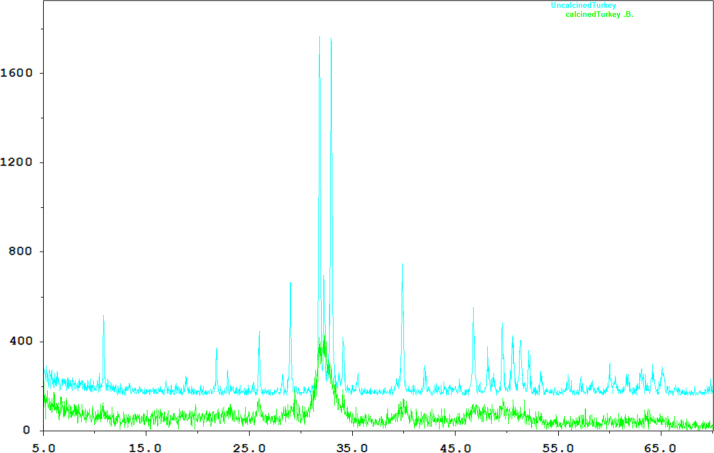
XRD analysis of uncalcined WTB (in blue) and calcined WTB (in green) catalyst.

**Fig. 2:**
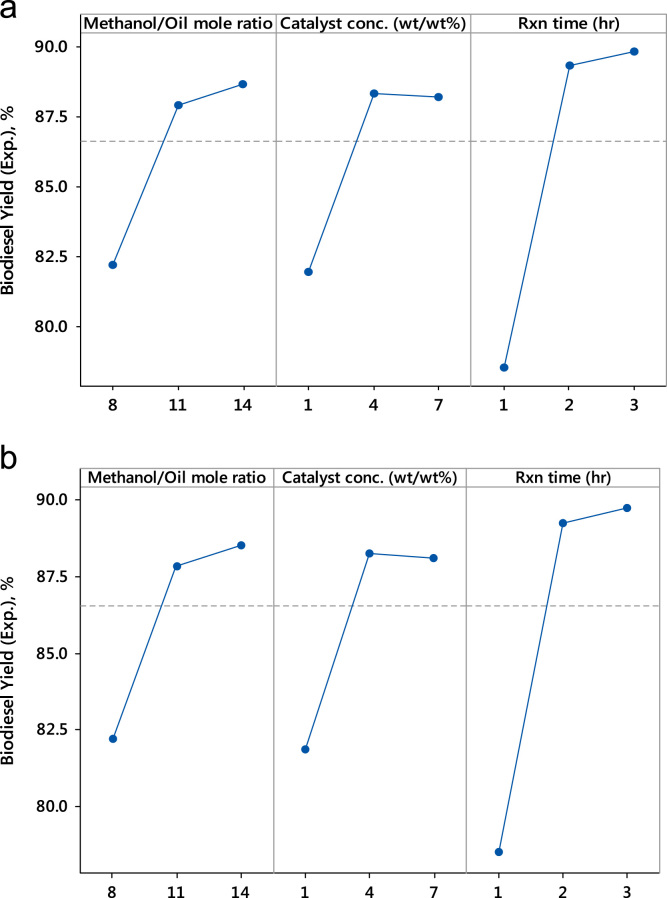
Main effects of the process variables: (a) CaO catalyst, (b) calcined WTB catalyst.

**Fig. 3:**
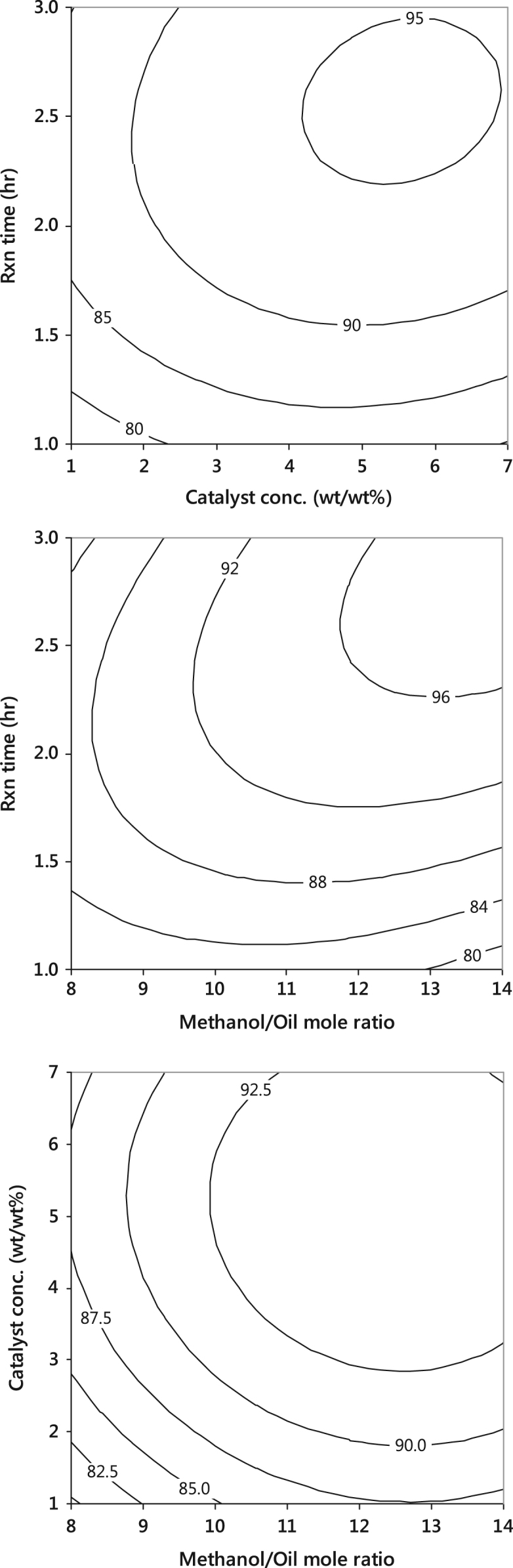
Interactive effects of the process variables on yield, using CaO catalyst.

**Fig. 4:**
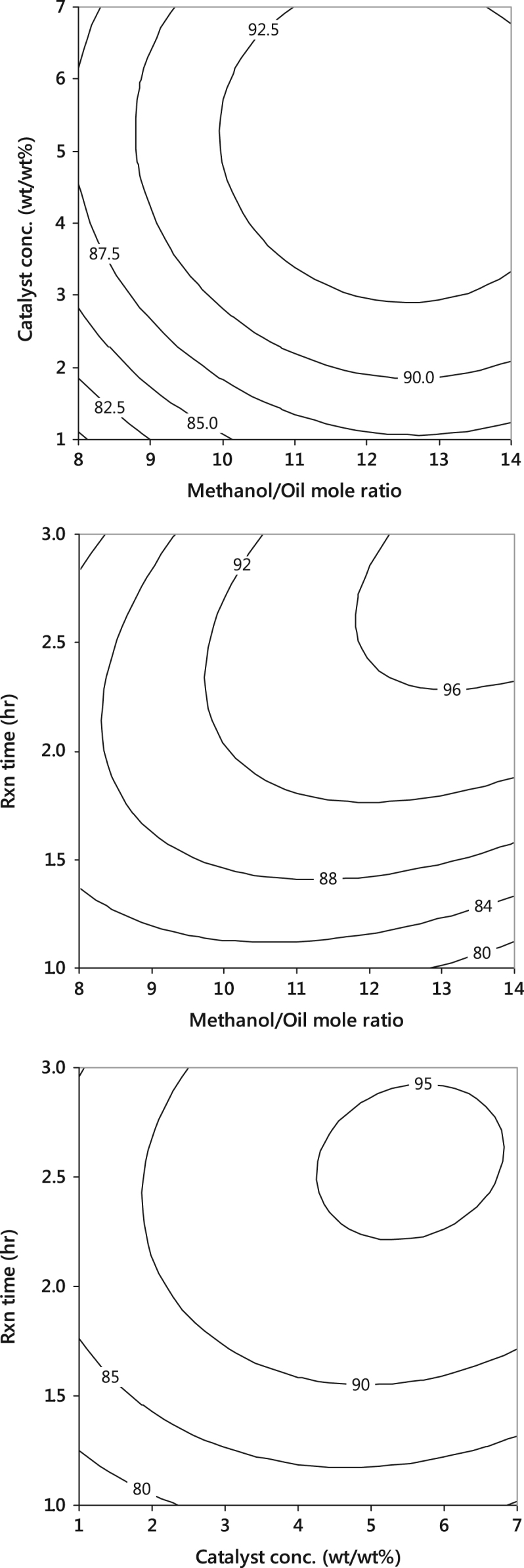
Interactive effects of the process variables on yield, using WTB catalyst.

Table 1 shows the data obtained from XRF analysis of CaO, uncalcined waste turkey bones and calcined waste turkey bones (used as catalyst). Fig. 1 shows the data obtained from XRD analysis of both the uncalcined WTB and calcined WTB. Table 2 shows the design of experiment, experimental biodiesel yield and calculated biodiesel yield data obtained from the transesterification process of PKO, using the conventional CaO and calcined WTB catalysts. Fig. 2 shows the main effects of the process variables (methanol/oil mole ratio, catalyst concentration and reaction time) on the biodiesel yields obtained. The interactive effects of the process variables (methanol/oil mole ratio, catalyst concentration and reaction time) on the biodiesel yields (using CaO catalyst) are shown is Fig. 3, while the interactive effects of the process variables (using calcined WTB catalyst) are shown in Fig. 4. Properties of the PKO biodiesel obtained are tabulated in Table 3. The suitable model equations obtained for the yields of biodiesel are shown in Eqs. (1), (2). Table 4 shows the analysis of variance (ANOVA) for CaO catalysed process, while Table 5 shows the analysis of variance (ANOVA) for WTB catalysed process.

**Table 1:** XRF analysis of CaO, uncalcined WTB and calcined WTB catalysts.

**Compounds**	**Composition of catalyst (%)**		
	**Calcium Oxide**	**Uncalcined WTB**	**Calcined WTB at 800** **°C**
CaO	99.230	62.335	65.599
P_2_O_5_	_	30.084	32.119
SiO_2_	_	2.882	1.766
MgO	0.110	0.640	0.296
Al_2_O_3_	_	0.647	0.465
SO_3_	_	0.454	0.120
Cl	–	0.085	0.100
K_2_O	_	0.103	0.128
TiO_2_	0.010	0.000	0.063
Cr_2_O_3_	–	0.002	0.000
Mn_2_O_3_	–	0.004	0.005
Fe_2_O_3_	0.110	0.158	0.184
ZnO	0.210	0.047	0.043
SrO	0.120	0.025	0.025
Na_2_O	0.020	0.214	0.356

**Table 2:** Design of experiment and biodiesel yields obtained.

Exptal. Run	Methanol/Oil mole ratio	Catalyst conc. (wt/wt%)	Rxn Time (hour)	Experimental % Biodiesel Yield using CaO	Calculated % Biodiesel Yield using CaO	Experimental % Biodiesel Yield using WTB	Calculated % Biodiesel Yield using WTB
1	8	1	2	78.65	79.57	73.00	72.94
2	14	1	2	87.56	86.68	84.00	83.54
3	8	7	2	85.90	86.60	85.30	85.74
4	14	7	2	93.23	92.13	87.20	87.23
5	8	4	1	82.30	80.22	78.00	77.67
6	14	4	1	78.00	77.54	74.20	74.27
7	8	4	3	82.20	82.48	77.90	77.81
8	14	4	3	95.90	97.80	93.00	93.31
9	11	1	1	75.56	76.54	72.89	73.26
10	11	7	1	78.50	79.70	82.30	82.17
11	11	1	3	86.10	84.731	83.40	83.51
12	11	7	3	95.20	94.04	91.50	91.11
13	11	4	2	94.30	93.30	90.90	90.62
14	11	4	2	93.46	93.30	91.00	90.63
15	11	4	2	92.40	93.30	90.00	90.62

**Table 3:** Properties of the biodiesel obtained.

Property	Unit	Method	PKO Biodiesel using CaO	PKO Biodiesel using WTB catalyst	ASTM Standard Values
Density (at 20 °C)	g/ml	ASTM D4052	0.882	0.876	0.860–0.890
Viscosity (at 40°)	mm^2^/s	ASTM D445	3.74	3.81	1.9–6.0
Cloud point	°C	ASTM D240	6	6	(−3)–12
Pour Point	°C	ASTM D92	2	1	(−15)–13
Flash point	°C	ASTM D93	128	130	93 minimum
Acid Value	mgKOH/g	ASTM D664	0.32	0.35	0.8 max

**Table 4:** Analysis of Variance (ANOVA) for the CaO catalysed process.

Source	Df	Adj SS	Adj MS	F- Value	P-Value	Remarks
Model	9	689.660	76.629	20.28	0.002	Highly Significant
X_1_: Methanol-Oil mole ratio	1	82.176	82.176	1.74	0.006	
X_2_: Catalyst Concentration	1	77.875	77.875	20.60	0.006	
X_3_: Reaction time	1	253.575	253.575	67.09	0.000	
X_1_X_1_	1	36.540	36.540	9.67	0.027	
X_2_X_2_	1	56.328	56.328	14.90	0.012	
X_3_X_3_	1	117.486	117.486	31.09	0.003	
X_1_X_2_	1	0.624	0.624	8.04	0.701	
X_1_X_3_	1	81.000	81.000	0.17	0.006	
X_2_X_3_	1	9.486	9.486	21.43	0.174	Insignificant
Lack-of-Fit	3	17.084	0.5.695	6.28	0.140	Insignificant
Pure Error	2	1.813	0.907	–	–	

R-sq=0.9733, R-sq(adj)= 0.9253,

**Table 5:** Analysis of Variance (ANOVA) for the calcined WTB catalysed process.

Source	Df	Adj SS	Adj MS	F- Value	P-Value	Remarks
Model	9	695.212	77.246	247.71	0.000	Highly Significant
X_1_: Methanol/Oil mole ratio	1	73.205	73.205	234.75	0.000	
X_2_: Catalyst Concentration	1	136.208	136.208	436.79	0.000	
X_3_: Reaction time	1	184.416	184.416	591.38	0.000	
X_1_X_1_	1	92.415	92.415	296.36	0.000	
X_2_X_2_	1	39.13	39.130	125.48	0.000	
X_3_X_3_	1	87.046	87.046	279.14	0.000	
X_1_X_2_	1	20.702	20.702	66.39	0.000	
X_1_X_3_	1	89.302	89.302	286.37	0.000	
X_2_X_3_	1	0.429	0.429	1.38	0.294	Insignificant
Lack-of-Fit	3	0.953	0.318	1.05	0.523	Insignificant
Pure Error	2	0.607	0.303	–	–	

R-sq=0.9978, R-sq(adj)=0.9937,

## 2 Experimental design, materials and methods

Response surface experimental design (Box-Behnken method, Minitab 17 software) was used in the determination of the effects of the process variables (methanol/oil mole ratio, catalyst concentration and reaction time) on biodiesel yield. Materials and reagents used include waste turkey bones (WTB), palm kernel oil (PKO), Calcium Oxide (99% purity, Sigma-Aldrich UK), H_2_SO_4_ (98% purity, Sigma-Aldrich UK), methanol (99.8% purity, J.T Baker USA), Potassium hydroxide (96.5%, BDH AnalAR Inc.), sodium Hydroxide pellets (97.5%, BDH AnalAR Inc.), Isopropyl Alcohol (98% purity, J.T Baker USA) and Benzene (96% purity, J.T Baker USA). Equipment used include XRD and XRF spectrometers for the identification and quantification of the elemental composition of CaO, uncalcined and calcined WTB catalysts.

Waste turkey bones were boiled in hot water for 3 hours (the hot water was replaced every 30 min to remove impurities), dried and then crushed. Treated WTB was reduced to fine particulate size of <150 µm and then calcinated at 800 °C for 3 h to increase its catalytic activity by its conversion from Calcium phosphate hydroxide (Ca_10_P_6_O_26_H_2_) to CaO. X-ray diffraction (XRD) and X-ray fluorescent (XRF) analysis of the analytical grade CaO, uncalcined and calcined WTB were carried out to establish their elemental chemical composition. X-ray fluorescence (XRF) (Thermo Scientific ARL OP-TIM’X 166) gave the composition of both the calcined and uncalcined WTB catalystS (Table 1).

X-ray Diffraction (XRD) analysis involved Mac science X-ray diffraction system (MXP3A-HF) with CuKα X-ray source (λ=0.15 nm and k=1.5406Å) operated at 30 mA and 40 kV. The diffractograms were recorded in the 2 h ranges of 5–70 with a 2 h step size of 0.03. This was done to determine the diffraction pattern of the finely grounded calcined and uncalcined waste turkey bones (WTB) catalyst. For the uncalcined WTB, major peaks were observed at 2θ=32.0° and 34.5°, other peaks were noticed at 2θ=29.0°, 34.0°, 40.0°, and 46.5° (Fig. 1). These peak values were the characteristics of CaO and Ca_10_P_6_O_26_H_2_. While the peak for the calcined WTB catalyst was measured at 2θ=32.0° and 33.0°, the main peak values characteristics of calcium oxide [1].

The procedures followed in the experimental production of PKO biodiesel were clearly stated in our previous work [2], [3]. The transesterification reaction between the triglyceride of palm kernel oil (PKO) and methanol was carried out at a constant agitation speed of 600 rpm and temperature of 65 °C, with varied methanol to oil molar ratio (8–14), catalyst concentration (1–7 wt/wt%) and the reaction time (1–3 h) (Table 2, Table 3 and Fig. 2, Fig. 3, Fig. 4).

Models that established relationship between the response (biodiesel yields) and the process parameters were formulated, using Minitab 17 software (Eqs. (1), (2)). Fitness and suitability of the predicted models were statistically analyzed using ANOVA test. The results gave high level of accurate prediction, with R^2^ values between 0.9253 and 0.9978 (Table 4, Table 5).

(1) $$\begin{array}{c} \mathrm{Biodiesel}\;{\mathrm{Yield}}_{\mathrm{CaO}}\\ =29.6+5.93X_{1}+3.97X_{2}+9.64X_{3}-0.350X_{1}^{2}-0.434X_{2}^{2}-5.64X_{3}^{2}\\ \;\;–0.044X_{1}X_{2}+1.500X_{1}X_{3}+0.513X_{2}X_{3} \end{array}$$

(2) $$\begin{array}{c} \mathrm{Biodiesel}\;{\mathrm{Yield}}_{\mathrm{WTB}}\\ =–5.38+11.099X_{1}+7.268X_{2}+7.33X_{3}–0.5559X_{1}^{2}–0.3617X_{2}^{2}\\ \;\;–4.855X_{3}^{2}–0.2528X_{1}X_{2}+1.575X_{1}X_{3}–0.1092X_{2}X_{3} \end{array}$$

where X_1_=Methanol/Oil mole ratio, X_2_=Catalyst concentration, X_3_=Reaction time
