# Supplementary material for: Data on PKO biodiesel production using CaO catalyst from Turkey bones
Source: Data Brief. 2018 May 23;19:789–97. doi: 10.1016/j.dib.2018.05.103 (PMC5997948; doi:10.1016/j.dib.2018.05.103)
Supplement: Supplementary file 1 — Supplementary material. [file mmc1.docx]

Chemical Engineering Department

Covenant University,

Nigeria

April 26, 2018

Dear Editor,

Data In Brief

**CONFLICT OF INTEREST**

The research article titled “**Data on PKO Biodiesel Production using CaO Catalyst from Turkey Bones”** is being submitted for publication in *Data In Brief*.

On behalf of all the authors, I declare that the manuscript has not been published in another publication and it is not being submitted simultaneously to another journal.

Forwarded for submission are the manuscript and conflict of interest form.

Thank you

Yours faithfully,

**Ayoola A. Ayodeji**

***Corresponding Author***
